# Myofascial Induction Therapy Improves the Sequelae of Medical Treatment in Head and Neck Cancer Survivors: A Single-Blind, Placebo-Controlled, Randomized Cross-Over Study

**DOI:** 10.3390/jcm10215003

**Published:** 2021-10-27

**Authors:** Eduardo Castro-Martín, Noelia Galiano-Castillo, Carolina Fernández-Lao, Lucía Ortiz-Comino, Paula Postigo-Martin, Manuel Arroyo-Morales

**Affiliations:** 1 Department of Physical Therapy, Faculty of Health Sciences, University of Granada, 18016 Granada, Spain; eduardoc@ugr.es (E.C.-M.); noeliagaliano@ugr.es (N.G.-C.); paulapostigo@ugr.es (P.P.-M.); marroyo@ugr.es (M.A.-M.); 2 Sport and Health University Research Institute (iMUDS), 18016 Granada, Spain; 3 Instituto de Investigación Biosanitaria ibs. GRANADA, 18014 Granada, Spain; 4 ‘Cuídate’ Support Unit for Oncology Patients, 18016 Granada, Spain; 5 Department of Physical Therapy, Faculty of Health Sciences, Campus of Melilla, University of Granada, 52005 Melilla, Spain

**Keywords:** head and neck neoplasms, musculoskeletal manipulations, pain, range of motion

## Abstract

Head and neck cancer (HNC) is the sixth most common cancer worldwide. Yet, less than 60% of HNC survivors receive adequate therapy for treatment-related sequelae. The objective of this study was to determine the efficacy of myofascial induction therapy (MIT) in improving cervical and shoulder pain and range of motion, maximal mouth opening, and cervical muscle function in HNC survivors. This crossover, blinded, placebo-controlled study involved 22 HNC survivors (average age 56.55 ± 12.71) of which 13 were males (59.1%) who received, in a crossover fashion, both a single 30-min session of MIT in the form of manual unwinding and simulated pulsed shortwave therapy (placebo), with a 4-week washout interval between the two. Cervical and shoulder pain (visual analogue scale) and range of motion (cervical range of motion device and goniometer), maximum mouth opening (digital caliper), and cervical muscle function (deep cervical flexor endurance test) were measured before and after the treatment and placebo sessions. A single session of MIT improved cervical and affected side shoulder pain, cervical range of motion, maximum mouth opening, and cervical muscle function. The associated effect sizes ranged from moderate to large. The present study suggests that MIT, in the form of manual unwinding, improves cervical (−3.91 ± 2.77) and affected-side shoulder (−3.64 ± 3.1) pain, cervical range of motion (flexion: 8.41 ± 8.26 deg; extension: 12.23 ± 6.55; affected-side rotation: 14.27 ± 11.05; unaffected-side rotation: 11.73 ± 8.65; affected-side lateroflexion: 7.95 ± 5.1; unaffected-side lateroflexion: 9.55 ± 6.6), maximum mouth opening (3.36 ± 3.4 mm), and cervical muscle function (8.09 ± 6.96 s) in HNC survivors.

## 1. Introduction

Head and neck cancer (HNC) refers to any malignant tumor arising above the thoracic inlet and below the base of the skull. It therefore includes the oral cavity, pharynx, larynx, salivary glands and sinonasal cavities [1]. HNC is the sixth most common cancer worldwide; in 2019 more than 550,000 new cases were diagnosed. Its prevalence in men is twice that seen in women [2].

Tumor size, location and stage, guide the decision to treat via surgery and/or radiotherapy and chemotherapy; more advanced tumors commonly require combined treatments [3]. Surgical treatment involves tumor resection, plus neck dissection if the lymph nodes are affected. This procedure can leave sequelae such as pain and lack of mobility, leading to neck and shoulder dysfunction [4]. For its part, cumulative radiotherapy can generate fibrosis, soft tissue necrosis and osteoradionecrosis [5], and chemotherapy can induce sensory and motor disorders [6]. The risk of developing significant functional deficiencies is even higher with concurrent chemoradiotherapy [7].

Treatment-induced pain in the neck and shoulder is common in survivors of HNC. In fact, some 70% of patients are affected by the end of treatment, as are over 35% at six months post-completion [8], and indeed over the first three years after diagnosis [9]. This pain is mainly considered nociceptive, but it may also be neuropathic in origin and involve peripheral and central sensitization processes [10]. Similarly, motor function in this region and in any treated adjacent region may worsen. For example, neurological damage and fibrotic changes may appear at surgery and radiotherapy sites, resulting in a reduction in cervical and shoulder range of motion (ROM) and maximum mouth opening (MMO) (including trismus), and causing difficulty in activating certain muscle groups [11,12]. The latter has been described in survivors of breast cancer, in whom altered patterns of cervical muscle activation were detected during functional upper limb tasks [13]. Yet, despite all this being known, less than 60% of all survivors of HNC receive adequate therapy for these sequelae [14].

Physical therapy should be provided throughout cancer care [15]. Multiple body systems (cardiovascular, musculoskeletal, joints or nervous system) could be affected by cancer itself and medical treatment. In this sense, physical therapy is helpful and could contribute to address these harmful effects [16]. In particular, manual therapy has been associated with improved blood circulation, reduced muscle spasms, increased ROM, the release of connective tissue adhesions, reduced pain and restoring mobility [17,18]. Specifically, in survivors of HNC manual therapy showed no specific adverse outcomes [19], reporting adverse events similar to those experienced by patients without cancer [20].

Myofascial induction therapy (MIT) can assist in eliminating functional limitations by helping to restore general health and reducing or eliminating pain. Taking into account that the origin of sequelae could be multiple and so different areas could be damaged, MIT would be the most suitable manual technique to influence other areas at some distance, owing to its systemic effects (theory of tissue tensegrity) [21]. For example, it has been shown to reduce pain and improve cervical and shoulder ROM in patients with breast cancer [22,23,24,25]. In addition, it can help the function of scarred areas once the remodeling process is complete [26]. However, no evidence has been collected to show that MIT reduces pain resulting from medical treatment in survivors of HNC, and while one report suggests it can improve MMO and cervical muscle function, this did not involve patients with cancer [27]. Given this background, the aim of the present work was to determine the immediate effects of MIT on cervical and shoulder pain, cervical and shoulder ROM, MMO and cervical muscle function in survivors of HNC with neck/shoulder morbidity in compared with placebo pulsed shortwave therapy. We hypothesized that MIT would improve all outcomes described due to the fact that all of them are sequelae well described by survivors of HNC.

## 2. Materials and Methods

### 2.1. Study Design

This study was designed as a single-blind, placebo-controlled, cross-over trial (ClinicalTrials.gov NCT04145180). This type of design allows inter-individual variation in outcome measures to be taken into account [22,23] and adheres to the CONSORT 2010 statement “extension to randomized crossover trials” [28] (Supplementary Table S1).

### 2.2. Participants

Twenty-two survivors of HNC were recruited from the Oncology and Maxillofacial units of the Virgen de las Nieves Hospital (Granada, Spain) between November 2019 and March 2020. To be eligible for inclusion, patients had to: (1) have had a diagnosis of HNC (stage I-IV) [29]; (2) be between 25 and 85 years of age; (3) have completed adjuvant therapy, and (4) perceive pain at a value of >3 on a 0–10 visual analogue scale (VAS). Patients were excluded if they: (1) had a recurrent cancer, (2) had suffered trauma or surgery in the oro-facial, cervical, thoracic or upper limb areas not related to cancer in the last 6 months, or (3) did not have medical clearance to participate.

**Figure 1:**
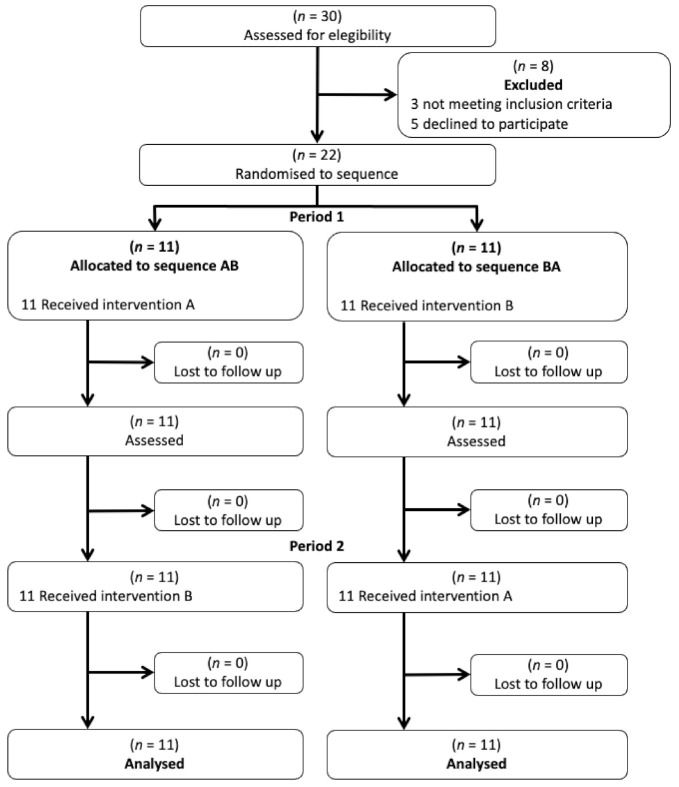
Flowchart according to CONSORT statement for the report of randomized trials.

A total of 22 patients were randomized (Figure 1). Thirteen of the 22 patients were male (59.1%) and 9 female (40.9%). Many patients were diagnosed with stage IV disease (40.9%). In total, some 95.4% of the subjects underwent surgery, 95.5% underwent radiotherapy and 68.2% underwent chemotherapy. The mean time elapsed since the diagnosis of cancer was 26.33 months. According to the Fonseca Anamnese Index [30], three patients (13.6%) had no temporomandibular disorder (TMD), 4 (18.2%) had mild TMD, 6 (27.3%) had moderate TMD, and 9 (40.9%) had severe TMD. Table 1 shows the remaining demographic and clinical data collected. No adverse effects were recorded.

### 2.3. Sample Size

The required sample size—for 80% power to detect a mean difference of 1.5 pain VAS points with a standard deviation of 3 points, a type 1 error (α) of 7.5%, and a type 2 error (β) of 20%—was calculated as previously described [22] and using the EPIDAT 3.1 software (Xeral de Saúde Pública, La Coruña, Spain). This determined the need for 20 patients in total; 22 were recruited to account for possible dropouts. The total trial finished once the sample size was reached.

### 2.4. Randomization to Treatment/Control Group, and Treatment Cross-Over

**Figure 2:**
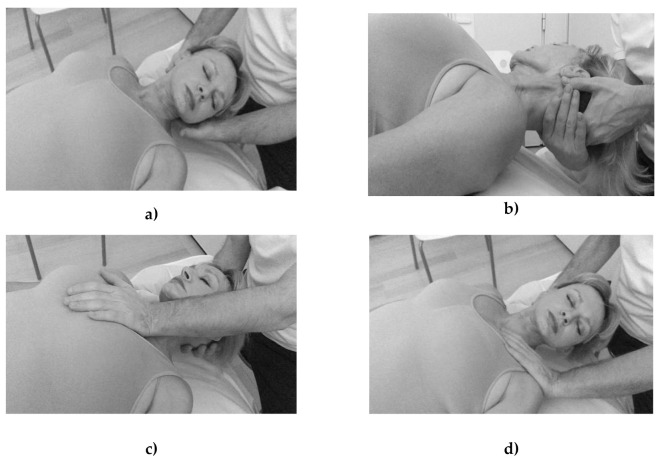
(**a**–**d**). Myofascial unwinding description over the neck area.

All patients were subject to one session of MIT in the form of myofascial unwinding (MU) for 30 min, and to mock pulsed shortwave therapy (placebo) for 30 min, separated by a four-week wash-out interval before cross-over. All interventions were performed by the same physical therapist in a physical therapy laboratory at the Health Science Faculty, University of Granada, Spain. The order of MIT/placebo treatment was decided by coin flipping (performed by a clinician blinded to the study). MU was performed as previously described [22] (Figure 2a–d); this is a safe and effective technique for restoring tissue mobility and function [22]. With the patient in the supine position, the physical therapist lifted the head, applied gentle and sustained traction, and moved the head as previously described [22]. The mock pulsed shortwave therapy for the placebo treatment was administered as previously described [22,23]. All sessions were conducted at the same time of day at 20–22 °C in a physical therapy laboratory.

### 2.5. Outcome Measures

Values for all measured variables were recorded immediately before and after the treatment and placebo sessions.

#### 2.5.1. Pain

The 0–10 VAS mentioned above was used to evaluate perceived pain in the cervical and shoulder regions. This scale has been widely used and has been demonstrated to be reliable and valid. It is commonly used to measure pain in different populations, including survivors of HNC [31].

#### 2.5.2. Range of Motion

To determine the cervical ROM (which includes the variables flexion, extension, affected-side rotation, unaffected-side rotation, affected-side lateroflexion and unaffected-side lateroflexion), patients were placed in a sitting position with their feet on the floor. A cervical range of motion (CROM) device (Performance Attainment Associates, Spine Products, Roseville, MN, USA) was used for this measurement. The intraclass correlation coefficient (ICC) for the CROM device ranges from 0.89 to 0.98 for the cervical ROM variables. Test-retest reliability of measurements was assessed, as well as its standard error of measurement and minimal detectable change, with 2 measures taken on 2 separate days spaced 48 h apart [32].

A plastic universal goniometer with two adjustable overlapping arms was used to measure shoulder ROM, including active flexion, abduction, and external and internal rotation (all for both the affected and unaffected sides). The ICC is ≥0.94 for the assessed shoulder joint movements. Intra-rater (2 measurements performed with a lapse of 24 h in between) and inter-rater reliability (2 measurements performed on the same day) have been evidenced, as well as its standard error of measurement and the minimal detectable change [33].

#### 2.5.3. Maximum Mouth Opening

Patients, sitting in an upright position, were requested to open the mouth as widely as possible without pain, and the distance between the incisal margins of the central maxillary and mandibular incisors was measured using an ABS Holex digital caliper (Hoffmann-Group, Munich, Germany) (resolution 0.01 mm, nominal capacity 150 mm, accuracy of ±0.02 mm). An ICC of 0.85–0.96 has been reported for this test [34].

#### 2.5.4. Cervical Muscle Function

**Figure 3:**
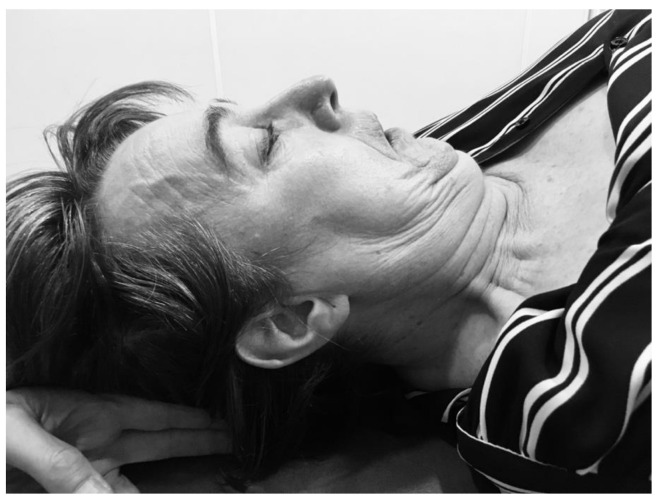
Deep cervical flexor endurance test description over the neck area.

The activity of the deep cervical flexor muscles was recorded using the deep cervical flexor endurance test (DCFET) (Figure 3). Patients were positioned supine on the treatment table. The therapist placed a hand under the patient’s head and requested that he/she execute a cervical flexion by making a “double chin” and lifting the head as little as possible to lose contact with the therapist’s hand. Time was measured from the moment the patient started the movement until: (1) the movement was lost (2) the head touched the therapist’s hand for more than a second, (3) the subject began to feel pain. An ICC of 0.82–0.91 has been reported for this test [35].

### 2.6. Statistical Analysis

Data were expressed as means and standard deviations (continuous data) or frequencies and percentages (categorical data). The normality of all variables was tested using the Kolmogorov–Smirnov test. The values of non-parametric variables were logarithmically transformed before use. Baseline differences for continuous variables between the MIT and placebo sessions were analyzed using the Student t-test or Mann Whitney U test as appropriate; the Chi2 test or Fisher’s exact test was used for categorical variables. To examine the effects of the MIT and placebo treatments, two-way repeated measures analysis of variance (ANOVA) was performed, using the treatments as the between-group variable, and time (pre–post treatment) as the within-group variable. If an interaction was found to have a significant influence, pairwise comparison was performed to determine whether the between-group difference in score change was also significant. Pairwise comparisons were corrected by multiplying the obtained *p* value by 4 (i.e., for 2 treatments at 2 different times). Effect sizes were calculated as Cohen’s d values. Significance was set at *p* < 0.05. Wherever possible, the results were examined in terms of clinically meaningful change. All calculations were made using SPSS software v.25.0 (IBM Statistics, Armonk, NY, USA).

### 2.7. Ethics Statement

The study was approved by the Ethics Committee of the University of Granada (CEi-GRANADA Ref: 0045-N-16) and was performed according to the guidelines established by the Helsinki Declaration and Law 14/2007 on Biomedical Research. All subjects received written information regarding the study and gave their informed consent to be included.

## 3. Results

### 3.1. Effects of MIT on Cervical and Shoulder Pain

The interaction time (i.e., pre or post treatment) x treatment had a significant, positive influence on cervical (F = 5.310; *p* = 0.026) and affected-side shoulder (F = 13.457; *p* = 0.001) pain. However, it had no influence on the unaffected shoulder (F = 0.025; *p* = 0.876) (Table 2). Pairwise comparisons revealed a significant reduction in the cervical (−3.91 points) and affected-side shoulder (−3.64 points) pain scores after the MIT session (all *p* < 0.004), and in cervical pain (−2.09) after the placebo session (*p* < 0.004), although the latter change did not reach the minimum required to be clinically meaningful (3 points) [36]. After the MIT session, 15 patients (68.2%) saw their scores surpass this threshold with respect to cervical pain, and 16 (72.7%) with respect to affected-side shoulder pain. The effect size of MIT was moderate for cervical pain (d = −0.70, 95%CI −1.29; −0.08) and large for affected-side shoulder pain (d = −1.11, 95%CI −1.72; −0.45).

### 3.2. Effects of MIT on Cervical and Shoulder Range of Movement

The interaction time x treatment had a significant, positive influence on all cervical ROM variables: flexion (F = 10.589; *p* = 0.002), extension (F = 53.850; *p* < 0.001), rotation on the affected side (F = 22.471; *p* < 0.001), rotation on the unaffected side (F = 25.217; *p* < 0.001), lateral flexion on the affected side (F = 25.217; *p* < 0.001) and lateral flexion on the unaffected side (F = 9.764; *p* = 0.003) (Table 3). Moreover, pairwise comparisons indicated a significant increase in the values of all cervical ROM variables following MIT (all *p* < 0.004); after the placebo session, values were either maintained or reflected a worsening. All changes in cervical ROM values surpassed the minimum threshold for clinically meaningful change (reference range of 3.6–6.5 degreesº) [32]. The effect size of MIT on the cervical ROM variables was always large: flexion (d = 0.98, 95%CI 0.34–1.59), extension (d = 2.21, 95%CI 1.43–2.92), affected-side rotation (d = 1.43, 95%CI 0.74–2.06), unaffected-side rotation (d = 1.45, 95%CI 0.76; 2.09), affected-side lateral flexion (d = 1.51, 95%CI 0.82–2.15), unaffected-side lateral flexion (d = 1.45, 95%CI 0.76–2.09).

The interaction time x treatment also had a significant, positive influence on the affected-side external rotation (F = 4.098; *p* = 0.049). No such influence was seen with respect to flexion, abduction and internal rotation for either side, or unaffected-side external rotation (all *p* > 0.05) (Table 4). In pairwise comparisons, no significant difference was seen in affected-side external rotation (*p* > 0.05); indeed, the minimum threshold for clinically meaningful change for shoulder ROM (reference range of 14–24 degrees) was not reached after either the MIT or placebo sessions [37].

### 3.3. Effects of MIT on Maximum Mouth Opening and Cervical Muscle Function

The interaction time x treatment had a significant influence on MMO (F = 17.153; *p* < 0.001) (Table 5). Pairwise comparisons revealed that after MIT, MMO improved significantly (score change +3.36 mm; *p* < 0.004), but not after the placebo session (score change −0.36). The same interaction also had a significant influence on cervical muscle function (F = 25.081; *p* < 0.001) (Table 5). In addition, pairwise comparisons showed a significant improvement in cervical muscle function after the MIT session (score change +8.09 s; *p* < 0.004); after the placebo session the score became slightly worse (score change −0.63 s). However, the minimum change in neck pain required to be clinically meaningful (19.05 s in the DCFET [38]) was not surpassed. The effect size for MIT was large, both for MMO (d = 1.25 95%CI 0.58; 1.87) and cervical muscle function (d = 1.51 95%CI 0.82; 2.15).

## 4. Discussion

The present results show that a single session of MIT in the form of MU improves pain, cervical ROM, MMO and cervical muscle function in survivors of HNC, with the effect size on these variables ranging from moderate to large. A single session does not, however, improve shoulder ROM in these patients.

Following the MIT session, the patients experienced a significant reduction in pain in the cervical area and the affected-side shoulder, with most showing a clinically meaningful difference (an improvement of >3 points) for both impairments (68.2% and 72.7%, respectively) [36]. The placebo session also led to a significant reduction in pain in the cervical region, perhaps due to some relaxation that the patients interpreted as a reduction in pain, but this was not clinically meaningful. Some studies report a single session of manual therapy to be beneficial in terms of pain relief, including MIT in patients with cervical or shoulder pain [22,23,24]. Fascial induction is used to treat fascial restriction and to restore tissue function [39], which might aid in pain reduction. Certainly, different massage techniques, including MIT techniques, have been used to treat patients with cancer to alleviate pain [22], and a systematic review indicates that different types of massage provide a safe and effective treatment for pain compared to no-treatment in such patients [40]. The present results would seem to endorse the idea that survivors of HNC also benefit from manual massage.

The single session of MIT also improved cervical ROM (all variables), with values surpassing the threshold of clinical significance [32]. MIT protocols have been shown effective in treating restrictions affecting the neck and shoulder in patients with cancer [22,24]. As suggested in our previous work [22,23], MU could promote a neurobiological response through stimulation of intrafascial mechanoreceptors, leading to the functional improvement of affected tissues. It should be noted that the present single session of MIT provided no benefit in terms of shoulder ROM.

The single MIT session significantly improved MMO. It is known that the range of mouth opening measured should include pain-free MMO for the diagnosis of mandibular disorders [41]. Indeed, this improvement was clinically meaningful: the mean MMO for the MIT-treated patients was above 35 mm, the upper threshold for trismus [42]. Many survivors of HNC experience difficulty in opening their mouths. Some authors have reported intraoral myofascial release to be effective in treating mouth opening in subjects with temporomandibular disorders [43], but to our knowledge no previous study has examined treating survivors of HNC via MIT. In the present work, one session of MIT in the form of MU focused on the cervical region, increased MMO. This may be explained by the dynamic biomechanical relationship that exists between the cervical spine and the temporomandibular joint during active mouth opening [44]. It has been reported that manual therapy of the cervical region increases active mouth opening in patients with temporomandibular disorders [45]. Suboccipital myofascial induction led to an immediate increase in pressure pain thresholds over latent trigger points in the masseter and temporalis muscles and an increase in MMO in non-oncological subjects [27]. Alternatively, stimulation in the cervical region might activate descending inhibitory pathways involving the cervical and trigeminal areas; via the trigemino-cervical nucleus, this might then improve cervical and oro-facial muscle behavior by ameliorating mandibular dynamics. MU might also generate changes in positional afferent inputs into the cervical spine, and thus improve the biomechanical relationship between the latter and the temporomandibular joint, increasing mouth opening [45].

The present results also show the effectiveness of the MIT technique used in increasing cervical muscle endurance (as determined by DFCET). In agreement with this, other authors have reported the effectiveness of manual therapy in improving isometric cervical strength in subjects with mechanical neck pain [46]. It is likely that the cervical muscle dysfunction that occurs in many patients who have survived HNC is related to structural changes [47]. The reduced neck pain in the present patients after MIT suggests this treatment may improve the isometric strength of deep cervical flexors. However, the improvement in muscle endurance did not reach the threshold of clinically meaningful change established for neck pain [38]. It may be that a single session of MIT is insufficient. More treatment sessions might be required to achieve the desired results.

The current work has several limitations. Firstly, the sample size, although sufficiently large for the present work, was small. A larger sample may have reduced interindividual differences. Secondly, only one treatment session was provided. More treatment sessions might have improved the results obtained. The fact that an improvement in pain was recorded for the affected-side shoulder, but not that shoulder’s ROM, suggests the results should be interpreted with caution. Further, all treatment was provided by the same physical therapist; while this may offer certain advantages in the research setting, it should be borne in mind that different practitioners might obtain different results in the clinical setting. Further experiments with more practitioners might throw light on the suitability of MIT in a setting more similar to routine clinical practice. To avoid all these limitations, future research should focus on performing studies with larger sample sizes, longer interventions and different physical therapists to evidence and clarify the effects of MIT in survivors of HNC.

## 5. Conclusions

The present results show the effectiveness of a single session of MIT in improving pain, cervical ROM, MMO and cervical muscle function, but not shoulder ROM, in survivors of HNC. Future work should involve more sessions to determine whether the results can be improved, as well as more physical therapists in order to better simulate the clinical setting.

## Figures and Tables

**Table 1:** Baseline demographic and clinical data (*n* = 22).

Age (y)	56.55 (12.71)
Gender Male Female	13 (59.1) 9 (40.9)
Alcohol consumption None Monthly Weekly Daily	12 (54.5) 4 (18.2) 4 (18.2) 2 (9.1)
Smoking habits Non smoker Ex-smoker Smoker	8 (36.4) 11 (50) 3 (13.6)
Time since diagnosis (months)	26.33 (16.68)
Affected-side Right Left	11 (50) 11 (50)
Cancer stage I II III IV	5 (22.7) 3 (13.6) 3 (13.6) 9 (40.9)
Surgery None MRND RND	1 (4.5) 9 (40.9) 12 (54.5)
Radiotherapy No Yes	1 (4.5) 21 (95.5)
Chemotherapy No Yes	7 (31.8) 15 (68.2)
Fonseca None Light Moderate Severe	3 (13.6) 4 (18.2) 6 (27.3) 9 (40.9)

Values are mean ± SD; or frequencies (%) at baseline. MRND: Modified radical neck dissection; RND: Radical neck dissection; y: years.

**Table 2:** Pre-intervention, post-intervention, and change scores for VAS (*n* = 22).

VAS	MIT Session	Placebo Session	*p* Value *
VAS cervical Pre-intervention Post-intervention Pre–post change score	5.32 ± 2.43 1.41 ± 2.30 −3.91 ± 2.77 †	5.68 ± 2.47 3.59 ± 2.70 −2.09 ± 2.45 †	*p* = 0.026
VAS affected-side shoulder Pre-intervention Post-intervention Pre–post differences	4.41 ± 2.93 0.77 ± 1.92 −3.64 ± 3.1 †	4.55 ± 2.89 3.95 ± 3.06 −0.59 ± 2.36	*p* = 0.001
VAS unaffected-side shoulder Pre-intervention Post-intervention Pre–post differences	1.55 ± 2.80 0.36 ± 1.33 −1.18 ± 2.34	2.32 ± 3.19 1.27 ± 2.58 −1.05 ± 3.33	*p* = 0.876

Values are mean ± SD at pre-intervention and post-intervention. The mean difference ± SD for pre–post change score. * Interaction time x treatment (ANOVA analysis). † Significant pairwise comparisons (*p* < 0.05). MIT: Myofascial induction therapy; VAS: Visual Analogue Scale.

**Table 3:** Pre-intervention, post-intervention, and change scores for cervical ROM (*n* = 22).

Cervical ROM (deg)	MIT Session	Placebo Session	*p* Value *
Flexion (deg) Pre-intervention Post-intervention Pre–post change score	47.18 ± 9.56 55.59 ± 9.06 8.41 ± 8.26 †	43.82 ± 13.12 43.41 ± 12.28 −0.41 ± 9.66	*p* = 0.002
Extension (deg) Pre-intervention Post-intervention Pre–post change score	43.86 ± 15.04 56.09 ± 14.93 12.23 ± 6.55 †	46.27 ± 15.27 41.95 ± 15.28 −4.32 ± 8.3	*p* < 0.001
Affected-side rotation (deg) Pre-intervention Post-intervention Pre–post change score	51.00 ± 15.84 65.27 ± 12.87 14.27 ± 11.05 †	51.73 ± 12.43 52.14 ± 16.38 0.41 ± 8.12	*p* < 0.001
Unaffected-side rotation (deg) Pre-intervention Post-intervention Pre–post change score	51.41 ± 15.25 63.14 ± 10.03 11.73 ± 8.65 †	51.45 ± 15.70 50.68 ± 12.46 −0.77 ± 8.56	*p* < 0.001
Affected-side lateroflexion (deg) Pre-intervention Post-intervention Pre–post change score	30.14 ± 9.04 38.09 ± 8.77 7.95 ± 5.1 †	31.64 ± 11.35 29.55 ± 8.76 −2.09 ± 7.88	*p* < 0.001
Unaffected-side lateroflexion (deg) ** Pre-intervention Post-intervention Pre–post change score	30.75 ± 9.39 38.32 ± 10.06 9.55 ± 6.6 †	32.73 ± 11.47 29.36 ± 9.51 −3.36 ± 10.71	*p* = 0.003

Values are mean ± SD at pre-intervention and post-intervention. The mean difference ± SD for pre–post change score. * Interaction time x treatment (ANOVA analysis). † Significant pairwise comparisons (*p* < 0.05). ** Logarithmically transformed. Deg: degrees; MIT: Myofascial induction therapy; VAS: Visual Analog Scale; ROM: range of motion.

**Table 4:** Pre-intervention, post-intervention, and change scores for shoulder ROM (*n* = 22).

Shoulder ROM (deg)	MIT Session	Placebo Session	*p* Value *
Affected-side flexion (deg) Pre-intervention Post-intervention Pre–post change score	152.86 ± 25.23 161.41 ± 23.28 8.55 ± 7.63	142.23 ± 29.51 144.86 ± 31.63 2.64 ± 13.45	*p* = 0.080
Unaffected-side flexion (deg) Pre-intervention Post-intervention Pre–post change score	161.05 ± 18.04 161.27 ± 21.15 3.61 ± 6.86	154.10 ± 20.78 154.32 ± 25.14 3.28 ± 9.86	*p* = 0.900
Affected-side abduction (deg) Pre-intervention Post-intervention Pre–post change score	138.09 ± 34.01 148.09 ± 35.73 10 ± 17.32	126.36 ± 43.28 131.27 ± 42.71 4.91 ± 19.24	*p* = 0.362
Unaffected-side abduction (deg) Pre-intervention Post-intervention Pre–post change score	151.43 ± 33.80 154.41 ± 27.30 5.10 ± 10.70	132.14 ± 41.23 139.36 ± 37.58 7.23 ± 26.18	*p* = 0.731
Affected-side external rotation (deg) Pre-intervention Post-intervention Pre–post change score	67.05 ± 24.08 74.05 ± 21.39 7 ± 11.20	73.18 ± 22.65 73.86 ± 22.63 0.68 ± 9.41	*p* = 0.049
Unaffected-side external rotation (deg) Pre-intervention Post-intervention Pre–post change score	72.76 ± 22.68 71.27 ± 22.97 0.48 ± 10.15	74.64 ± 18.75 73.36 ± 20.76 −1.27 ± 15.88	*p* = 0.671
Affected-side internal rotation (deg) Pre-intervention Post-intervention Pre–post change score	71.77 ± 15.55 76.55 ± 16.03 4.77 ± 8.04	67.32 ± 18.12 67.68 ± 18.34 0.36 ± 11.84	*p* = 0.156
Unaffected-side internal rotation (deg) Pre-intervention Post-intervention Pre–post change score	73.38 ± 14.92 70.73 ± 18.09 −0.71 ± 7.77	75.45 ± 19.20 67.73 ± 23.04 −7.73 ± 19.33	*p* = 0.130

Values are mean ± SD at pre-intervention and post-intervention. The mean difference ± SD for pre–post change score. * Interaction time x treatment (ANOVA analysis). Deg: degrees; MIT: Myofascial induction therapy; VAS: Visual Analog Scale; ROM: range of motion.

**Table 5:** Pre-intervention, post-intervention, and change scores for MMO and DCFET (*n* = 22).

	MIT Session	Placebo Session	*p* Value *
MMO (mm) Pre-intervention Post-intervention Pre–post change score	33.14 ± 10.96 36.5 ± 11.55 3.36 ± 3.4 †	30.05 ± 10.21 29.68 ± 11.33 −0.36 ± 2.50	*p* < 0.001
DCFET (s) Pre-intervention Post-intervention Pre–post change score	11.10 ± 7.95 19.19 ± 10.05 8.09 ± 6.96 †	9.70 ± 7.53 9.07 ± 7.86 −0.63 ± 4.26	*p* < 0.001

Values are mean ± SD at pre-intervention and post-intervention. The mean difference ± SD for pre–post change score. * Interaction time x treatment (ANOVA analysis). † Significant pairwise comparisons (*p* < 0.05). DCFET: Deep cervical flexor endurance test; s: seconds; mm: millimeters; MIT: Myofascial induction therapy; MMO: Maximal mouth opening.

## Data Availability

Not applicable.

## Supplementary Materials

The following are available online at https://www.mdpi.com/article/10.3390/jcm10215003/s1 Table S1: CONSORT checklist of information to include when reporting randomised crossover trials.

## References

[1] Cohen N., Fedewa S., Chen A.Y. (2018). Epidemiology and Demographics of the Head and Neck Cancer Population. Oral Maxillofac. Surg. Clin. N. Am..

[2] Aupérin A. (2020). Epidemiology of Head and Neck Cancers: An Update. Curr. Opin. Oncol..

[3] Shah J., Patel S., Singh B., Wong R. (2019). Head and Neck Surgery and Oncology.

[4] Gane E.M., Michaleff Z.A., Cottrell M.A., McPhail S.M., Hatton A.L., Panizza B.J., O’Leary S.P. (2017). Prevalence, Incidence, and Risk Factors for Shoulder and Neck Dysfunction after Neck Dissection: A Systematic Review. Eur. J. Surg. Oncol..

[5] Maghami E., Ho A. (2018). Multidisciplinary Care of the Head and Neck Cancer Patient.

[6] Baggi F., Santoro L., Grosso E., Zanetti C., Bonacossa E., Sandrin F., Massaro M.A., Tradati N., Simoncini M.C. (2014). Recupero Motorio e Funzionale Dopo Dissezione Latero-Cervicale Del Collo: Due Programmi Di Fisioterapia Precoce a Confronto. Acta Otorhinolaryngol. Ital..

[7] Huang J., Zhang J., Shi C., Liu L., Wei Y. (2016). Survival, Recurrence and Toxicity of HNSCC in Comparison of a Radiotherapy Combination with Cisplatin versus Cetuximab: A Meta-Analysis. BMC Cancer.

[8] Bossi P., Giusti R., Tarsitano A., Airoldi M., De Sanctis V., Caspiani O., Alterio D., Tartaro T., Alfieri S., Siano M. (2019). The Point of Pain in Head and Neck Cancer. Crit. Rev. Oncol./Hematol..

[9] Cramer J.D., Johnson J.T., Nilsen M.L. (2018). Pain in Head and Neck Cancer Survivors: Prevalence, Predictors, and Quality-of-Life Impact. Otolaryngol.-Head Neck Surg..

[10] Ortiz-Comino L., Fernández-Lao C., Castro-Martín E., Lozano-Lozano M., Cantarero-Villanueva I., Arroyo-Morales M., Martín-Martín L. (2020). Myofascial Pain, Widespread Pressure Hypersensitivity, and Hyperalgesia in the Face, Neck, and Shoulder Regions, in Survivors of Head and Neck Cancer. Support. Care Cancer..

[11] Moloney E.C., Brunner M., Alexander A.J., Clark J. (2015). Quantifying Fibrosis in Head and Neck Cancer Treatment: An Overview. Head Neck..

[12] Kraaijenga S.A.C., Oskam I.M., Van Der Molen L., Hamming-Vrieze O., Hilgers F.J.M., Van Den Brekel M.W.M. (2015). Evaluation of Long Term (10-Years+) Dysphagia and Trismus in Patients Treated with Concurrent Chemo-Radiotherapy for Advanced Head and Neck Cancer. Oral Oncol..

[13] Galiano-Castillo N., Fernández-Lao C., Cantarero-Villanueva I., Fernández-De-Las-Peñas C., Menjón-Beltrán S., Arroyo-Morales M. (2011). Altered Pattern of Cervical Muscle Activation during Performance of a Functional Upper Limb Task in Breast Cancer Survivors. Am. J. Phys. Med. Rehabil..

[14] Ringash J. (2015). Survivorship and Quality of Life in Head and Neck Cancer. J. Clin. Oncol..

[15] Pidlyskyj K., Roddam H., Rawlinson G., Selfe J. (2014). Exploring Aspects of Physiotherapy Care Valued by Breast Cancer Patients. Physiother.

[16] Morishita S., Tsubaki A., Suzuki T. (2017). Physical Therapy in Patients with Cancer. Clinical Physical Therapy.

[17] Bialosky J.E., Beneciuk J.M., Bishop M.D., Coronado R.A., Penza C.W., Simon C.B., George S.Z. (2018). Unraveling the Mechanisms of Manual Therapy: Modeling an Approach. J. Orthop. Sports Phys. Ther..

[18] Pinheiro da Silva F., Moreira G.M., Zomkowski K., Amaral de Noronha M., Flores Sperandio F. (2019). Manual Therapy as Treatment for Chronic Musculoskeletal Pain in Female Breast Cancer Survivors: A Systematic Review and Meta-Analysis. J. Manip. Physiol. Ther..

[19] Krisciunas G.P., Golan H., Marinko L.N., Pearson W., Jalisi S., Langmore S.E. (2016). A novel manual therapy programme during radiation therapy for head and neck cancer - our clinical experience with five patients. Clin. Otolaryngol..

[20] Krisciunas G.P., Vakharia A., Lazarus C., Taborda S.G., Martino R., Hutcheson K., McCulloch T., Langmore S.E. (2019). Application of Manual Therapy for Dysphagia in Head and Neck Cancer Patients: A Preliminary National Survey of Treatment Trends and Adverse Events. Glob. Adv. Health Med..

[21] Pilat A. (2003). Myofascial Therapies: Myofascial Induction.

[22] Castro-Martín E., Ortiz-Comino L., Gallart-Aragón T., Esteban-Moreno B., Arroyo-Morales M., Galiano-Castillo N. (2017). Myofascial Induction Effects on Neck-Shoulder Pain in Breast Cancer Survivors: Randomized, Single-Blind, Placebo-Controlled Crossover Design. Arch. Phys. Med. Rehabil..

[23] Castro-Martín E., Galiano-Castillo N., Ortiz-Comino L., Cantarero-Villanueva I., Lozano-Lozano M., Arroyo-Morales M., Fernández-Lao C. (2020). Effects of a Single Myofascial Induction Session on Neural Mechanosensitivity in Breast Cancer Survivors: A Secondary Analysis of a Crossover Study. J. Manip. Physiol. Ther..

[24] Serra-Añó P., Inglés M., Bou-Catalá C., Iraola-Lliso A., Espí-López G.V. (2019). Effectiveness of Myofascial Release after Breast Cancer Surgery in Women Undergoing Conservative Surgery and Radiotherapy: A Randomized Controlled Trial. Support. Care Cancer.

[25] Fourie W.J., Robb K.A. (2009). Physiotherapy Management of Axillary Web Syndrome Following Breast Cancer Treatment: Discussing the Use of Soft Tissue Techniques. Physiotherapy.

[26] Chamorro Comesaña A., Suárez Vicente M.D.P., Docampo Ferreira T., Pérez-La Fuente Varela M.D.M., Porto Quintáns M.M., Pilat A. (2017). Effect of Myofascial Induction Therapy on Post-c-Section Scars, More than One and a Half Years Old. Pilot Study. J. Bodyw. Mov. Ther..

[27] Oliveira-Campelo N.M., Rubens-Rebelatto J., Martín-Vallejo F.J., Alburquerque-Sendín F., Fernández-De-Las-Peñas C. (2010). The Immediate Efects of Atlanto-Occipital Joint Manipulation and Suboccipital Muscle Inhibition Technique on Active Mouth Opening and Pressure Pain Sensitivity over Latent Myofascial Trigger Points in the Masticatory Muscles. J. Orthop. Sports Phys. Ther..

[28] Dwan K., Li T., Altman D.G., Elbourne D. (2019). CONSORT 2010 Statement: Extension to Randomised Crossover Trials. BMJ.

[29] Patel S.G., Shah J.P. (2005). TNM Staging of Cancers of the Head and Neck: Striving for Uniformity Among Diversity. CA Cancer J. Clin..

[30] Diagnóstico Pela Anamnese da Disfunção Craniomandibular–ScienceOpen. https://www.scienceopen.com/document?vid=ca2c5ce5-8697-4e9b-85c9-88c70931a0da.

[31] Su T.L., Chen A.N., Leong C.P., Huang Y.C., Chiang C.W., Chen I.H., Lee Y.Y. (2017). The Effect of Home-Based Program and Outpatient Physical Therapy in Patients with Head and Neck Cancer: A Randomized, Controlled Trial. Oral Oncol..

[32] Audette I., Dumas J.P., Côté J.N., De Serres S.J. (2010). Validity and Between-Day Reliability of the Cervical Range of Motion (CROM) Device. J. Orthop. Sports Phys. Ther..

[33] Kolber M.J., Fuller C., Marshall J., Wright A., Hanney W.J. (2012). The Reliability and Concurrent Validity of Scapular Plane Shoulder Elevation Measurements Using a Digital Inclinometer and Goniometer. Physiother. Theory Pract..

[34] Rauch A., Schierz O. (2018). Reliability of Mandibular Movement Assessments Depending on TMD. Cranio-J. Craniomandib. Pract..

[35] Harris K.D., Heer D.M., Roy T.C., Santos D.M., Whitman J.M., Wainner R.S. (2005). Reliability of a Measurement of Neck Flexor Muscle Endurance. Phys. Ther..

[36] Lee J.S., Hobden E., Stiell I.G., Wells G.A. (2003). Clinically Important Change in the Visual Analog Scale after Adequate Pain Control. Acad. Emerg. Med..

[37] Muir S.W., Corea C.L., Beaupre L. (2010). Evaluating Change in Clinical Status: Reliability and Measures of Agreement for the Assessment of Glenohumeral Range of Motion. N. Am. J. Sports Phys. Ther..

[38] Lourenço A.S., Lameiras C., Silva A.G. (2016). Neck Flexor and Extensor Muscle Endurance in Subclinical Neck Pain: Intrarater Reliability, Standard Error of Measurement, Minimal Detectable Change, and Comparison With Asymptomatic Participants in a University Student Population. J. Manipulative Physiol. Ther..

[39] Pilat A., Liem T., Tozzi P.C. (2017). Myofascial Induction Therapy (MIT). Fascia in Osteopahty.

[40] Boyd C., Crawford C., Paat C.F., Price A., Xenakis L., Zhang W., Buckenmaier C., Buckenmaier P., Cambron J., Deery C. (2016). The Impact of Massage Therapy on Function in Pain Populations–a Systematic Review and Meta-Analysis of Randomized Controlled Trials: Part II, Cancer Pain Populations. Pain Med..

[41] Li D.T.S., Leung Y.Y. (2021). Temporomandibular Disorders: Current Concepts and Controversies in Diagnosis and Management. Diagnostics.

[42] Loh S.Y., Mcleod R.W.J., Elhassan H.A. (2017). Trismus Following Different Treatment Modalities for Head and Neck Cancer: A Systematic Review of Subjective Measures. Eur. Arch. Oto-Rhino-Laryngol..

[43] Kalamir A., Pollard H., Vitiello A., Bonello R. (2010). Intra-Oral Myofascial Therapy for Chronic Myogenous Temporomandibular Disorders: A Randomized, Controlled Pilot Study. J. Man. Manip. Ther..

[44] Zafar H., Nordh E., Eriksson P.O. (2000). Temporal Coordination between Mandibular and Head-Neck Movements during Jaw Opening-Closing Tasks in Man. Arch. Oral Biol..

[45] La Touche R., Fernández-De-Las-Peñas C., Fernández-Carnero J., Escalante K., Angulo-Díaz-Parreño S., Paris-Alemany A., Cleland J.A. (2009). The Effects of Manual Therapy and Exercise Directed at the Cervical Spine on Pain and Pressure Pain Sensitivity in Patients with Myofascial Temporomandibular Disorders. J. Oral Rehabil..

[46] Namvar H., Olyaei G., Moghadam B.A., Hosseinifar M. (2016). Effect of Myofascial Release Technique on Pain, Disability, Maximum Isometric Contraction of the Extensor Muscles, and Pressure Pain Threshold in Patients with Chronic Nonspecific Neck Pain: Double Blinded Randomized Clinical Trial. Int. J. Med. Res. Heal. Sci..

[47] Gane E.M., McPhail S.M., Hatton A.L., Panizza B.J., O’Leary S.P. (2019). Neck and Shoulder Motor Function Following Neck Dissection: A Comparison with Healthy Control Subjects. Otolaryngol.-Head Neck Surg..

